# Evaluation of Oral Mucosa Capillaries in Fibromyalgia Patients

**DOI:** 10.3390/biomedicines13112701

**Published:** 2025-11-03

**Authors:** Salvatore Nigliaccio, Davide Alessio Fontana, Francesca Pusateri, Emanuele Di Vita, Pietro Messina, Enzo Cumbo, Giuseppe Alessandro Scardina

**Affiliations:** Department of Precision Medicine in Medical, Surgical and Critical Care (Me.Pre.C.C.), University of Palermo, 90127 Palermo, Italy; salvo.nigliaccio@gmail.com (S.N.); davidealessiofontana@libero.it (D.A.F.); francescapusateri24@gmail.com (F.P.); emanueledivita95@gmail.com (E.D.V.); pietro.messina01@unipa.it (P.M.); enzo.cumbo@unipa.it (E.C.)

**Keywords:** fibromyalgia, oral mucosa, videocapillaroscopy, capillaries, microcirculation

## Abstract

**Background:** Fibromyalgia (FM) is a chronic pain syndrome characterized by widespread musculoskeletal pain, fatigue, unrefreshed sleep, and cognitive disturbances. Despite extensive research, its pathophysiology remains incompletely understood, and there are no validated biomarkers for diagnosis. Videocapillaroscopy is a non-invasive imaging technique that enables detailed visualization of microvascular structures and may provide insights into microcirculatory alterations associated with FM. **Methods:** Thirty patients with FM and 30 healthy controls underwent oral videocapillaroscopy at four sites: right and left buccal mucosa and upper and lower labial mucosa. Quantitative parameters, including capillary caliber, density, and length, were extracted using a validated neural-network-based software, while qualitative parameters, including visibility, orientation, and the presence of microhemorrhages, were assessed by the operator. **Results:** Capillary length was significantly reduced in fibromyalgia patients (297.49 ± 26.82 µm) compared to healthy controls (324.43 ± 37.59 µm; *p* = 0.002), and capillary orientation differed significantly between groups (*p* < 0.05). Capillary caliber, density, and visibility did not show statistically significant differences. **Conclusions:** These findings indicate subtle microvascular alterations in the oral mucosa of patients with fibromyalgia. Although the observed changes are not sufficient for diagnostic purposes or early detection, they provide preliminary evidence that videocapillaroscopy can detect microvascular features associated with FM in the oral mucosa. Further studies with larger cohorts and longitudinal designs are warranted to clarify the clinical relevance of these observations and to explore their potential association with symptom severity or disease progression.

## 1. Introduction

Fibromyalgia (FM) is a chronic pain disorder characterized by widespread musculoskeletal pain, persistent fatigue, non-restorative sleep, and cognitive impairment. Despite notable advances in recent years, important gaps in knowledge remain. Worldwide, the prevalence of fibromyalgia is estimated to range between 2% and 3% [1]. FM predominantly affects women, and although the revision of diagnostic criteria has adjusted the estimated female-to-male ratio from 9:1 to approximately 3:1, research continues to focus almost exclusively on female cohorts [2]. This imbalance limits a comprehensive understanding of sex-related differences in the disease. The etiopathogenesis of FM is still not fully clarified, leaving many clinical and pathophysiological questions unresolved [3,4]. Diagnosis remains largely clinical, as no universally validated or standardized instrumental or laboratory biomarker is currently available [5,6]. As a result, diagnostic processes are often complex and delayed, with substantial consequences for patients’ quality of life [7]. In this context, current research is increasingly directed toward identifying peripheral biomarkers and applying non-invasive techniques to objectively capture the pathophysiological alterations underlying the syndrome.

Although the pathophysiology of FM has not yet been fully elucidated, a growing body of evidence suggests that the syndrome is characterized by endothelial dysfunction [8,9] and low-grade inflammation [10,11]. Studies have consistently linked FM to objective peripheral vascular changes, including enhanced arterial stiffness [12] and increased oxidative stress [13], and to neurogenic inflammation [14] with increased proinflammatory cytokines [15]. Specifically, alterations in the release of vasoactive substances, such augmented Endothelin-1 (EDN1) [16], and reduced Nitric Oxide (NO) [17,18] bioavailability are observed, indicating an altered endothelium-related vasoconstriction. Given this strong rationale of systemic microvascular involvement, the microcirculation has previously been investigated in FM using techniques such as nailfold videocapillaroscopy [19], and ophthalmic microvascular studies [20]. The oral mucosa, a site known for its ease of access and unique capillary structure, offers a distinct and potentially highly sensitive window into systemic microvascular health.

**Figure 1:**
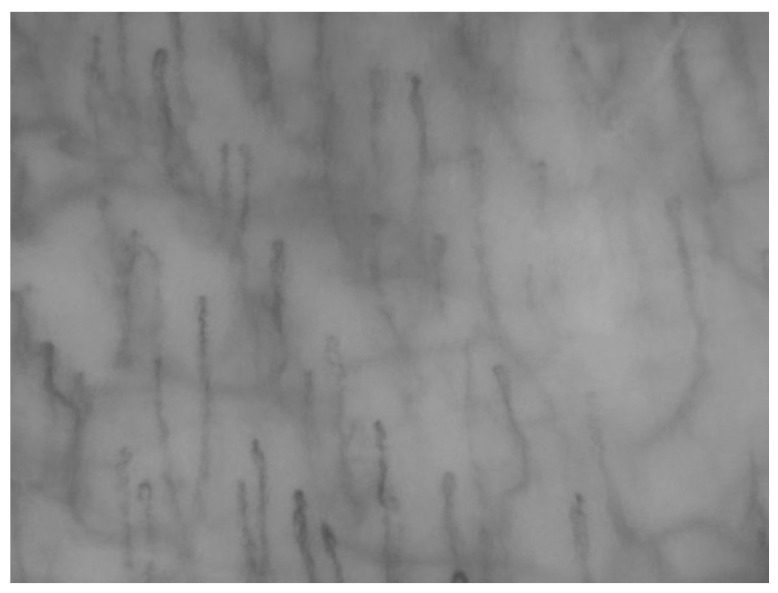
Illustrative example of a capillary field in the oral mucosa (left cheek).

Videocapillaroscopy is a non-invasive imaging technique that provides high-resolution visualization of the microvascular network (Figure 1) and has been increasingly employed to investigate subtle vascular changes in different clinical settings [21]. Alterations in the morphology of oral mucosal capillaries, both in quantitative and qualitative terms, have been reported in diseases of autoimmune origin [22,23] as well as in metabolic disorders [24]. Notably, microcirculatory abnormalities can sometimes be observed even in individuals who have not yet received a formal diagnosis, such as in the early stages of diabetes.

Within this framework, in the present study at this foundational stage, we aimed for data collection and the identification of specific microvascular patterns or signatures associated with Fibromyalgia, thus providing objective morphological evidence for the hypothesis of vascular involvement. Second, we aimed to verify the potential utility of oral videocapillaroscopy for early detection or risk stratification in FM, mirroring its established role in microangiopathies like systemic sclerosis and diabetic complications. To this end, videocapillaroscopic parameters obtained from a cohort of individuals with fibromyalgia were compared with those of healthy controls, with the aim of highlighting potential differences that could shed light on the pathophysiological mechanisms of the syndrome.

## 2. Materials and Methods

### 2.1. Study Design

This study was conducted at the Policlinico Universitario Paolo Giaccone in Palermo and involved a cohort of 60 volunteers, comprising 30 patients with fibromyalgia and 30 healthy controls. Written informed consent was obtained from all participants prior to enrolment and all procedures were conducted according to the Declaration of Helsinki. Each participant completed a comprehensive anamnesis questionnaire to verify eligibility according to the following criteria:

- Inclusion criteria: certified diagnosis of fibromyalgia established by a specialist using American College of Rheumatology (ACR) criteria for fibromyalgia.
- Exclusion criteria: conditions known to affect the microcirculation, including pregnancy, tobacco use, and alcohol or substance abuse.

To minimize confounding factors, all participants observed a resting period of at least 30 min between the anamnesis phase and the videocapillaroscopy procedure, during which they abstained from food, beverages (except water), and candy.

### 2.2. Demographics and Pathological/Pharmacological Anamnesis

The fibromyalgia cohort consisted entirely of female participants (*n* = 30), with a mean age of 53.6 ± 10.5 years (range: 29–69 years). The control group included 30 healthy individuals, of whom 20 were women and 10 were men, with a mean age of 44.8 ± 14.4 years (range: 23–65 years). While the control group included both sexes, no sex-related differences in the parameters analyzed have been reported to date.

Participants with fibromyalgia had received a confirmed diagnosis for an average of 7.6 years (range: 1–19 years). Among them, 22 out of 30 reported the use of anti-inflammatory drugs or corticosteroids, 12 out of 30 were taking muscle relaxants, and 10 out of 30 reported the use of non-smoked cannabis.

### 2.3. Data Collection and Videocapillaroscopy Analysis

Videocapillaroscopy examinations were performed using a Horus HS100 device, equipped with a 150× zoom lens, a 640 × 480 pixel resolution, a 120 FPS frame rate, and monochromatic imaging. The assessment focused on four oral sites:

- Right buccal mucosa
- Left buccal mucosa
- Lower labial mucosa
- Upper labial mucosa

For each site, a video of approximately 5s was recorded, consistently targeting the same anatomical landmarks to ensure the standardization and reproducibility of data collection.

Analysis of the videocapillaroscopic images was conducted using two complementary approaches. Quantitative parameters were extracted using a software developed at the University of Palermo in collaboration with the Unit of Odontostomatology [25]. This software, based on a specifically trained neural network, has been previously validated and provides numerical values for capillary diameter, density, and mean length, averaging measurements across all frames of the recorded video.

Qualitative parameters including capillary visibility, orientation, and the presence of microhemorrhages were assessed directly by the operator during the videocapillaroscopy procedure, allowing for real-time evaluation of structural and morphological features. However, the assessment of qualitative parameters was verified by a second expert operator who was completely blinded to the patient’s clinical status. Given the simple and objective morphological characterization of the non-parametric data used in this Brief Report, the secondary check revealed a 100% concordance between the two operators, confirming the high reliability and objectivity of the qualitative assessment.

### 2.4. Parameters Analyzed

- Parametric data:
  ○ Loop density: capillaries per mm^2^.
  ○ Loop caliber: mean of arteriolar and venular diameters in µm.
  ○ Loop length in µm.
- Non-parametric data:
  ○ Capillary visibility: clearly visible (1), poorly visible (2), or not visible (3).
  ○ Orientation in relation to the surface: parallel (A), perpendicular (B), or mixed (AB).
  ○ Presence of microhemorrhages and/or microaneurysms.

### 2.5. Statistical Analysis

All statistical analyses were performed using XLSTAT software (version 2024.4.0.1424, Lumivero) [26]. To address potential issues of pseudoreplication arising from multiple measurements taken within a single subject, we performed statistical comparisons using subject-level summary data. Specifically, all quantitative microvascular parameters (Length, Density, and Caliber) were averaged across all measured sites and frames for each individual patient. This resulted in a single mean value per parameter for each participant, which was then used in the independent two-sample t-tests to compare the Fibromyalgia and Control groups, with a two-tailed *p*-value < 0.05 considered statistically significant. In addition to *p*-values, effect sizes (Hedges’ g) were calculated to estimate the magnitude of group differences. Following the recent field-specific recommendations [27], thresholds of 0.1, 0.3, and 0.7 were interpreted as small, medium, and large effects, respectively.

Non-parametric data were analyzed using independence tests applied to contingency tables. The chi-square statistic and corresponding *p*-values were estimated through Monte Carlo simulation with 5000 iterations.

## 3. Results

The study included 30 patients with fibromyalgia and 30 healthy controls, with a total of 240 videocapillaroscopic images analyzed (Table 1).

### 3.1. Caliber

**Figure 2:**
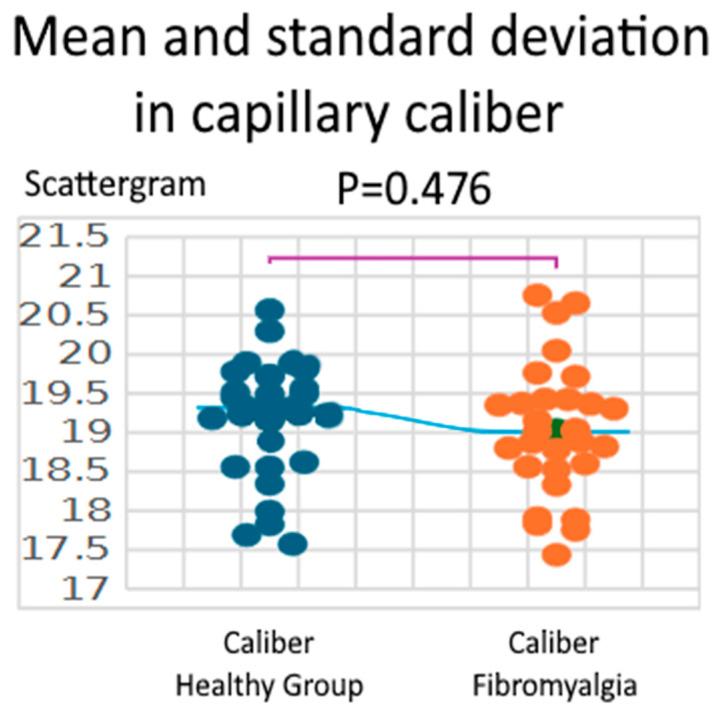
Comparison between healthy group and fibromyalgia Caliber.

Capillary caliber did not differ significantly between groups. Healthy controls showed a mean caliber of 19.18 ± 0.74 µm, while patients with fibromyalgia had a mean of 19.03 ± 0.83 µm. The Shapiro–Wilk test confirmed normal distribution in both groups (W = 0.940, *p* = 0.094 for healthy controls; W = 0.969, *p* = 0.508 for fibromyalgia). The independent two-sample t-test, assuming equal variances, revealed no significant difference between groups (t = 0.717, df = 58, *p* = 0.476; 95% CI: −0.26 to 0.56 µm). Although the difference was not statistically significant, the observed effect size (Hedges’ g = 0.185) suggests a small difference in capillary caliber (Figure 2).

### 3.2. Density

**Figure 3:**
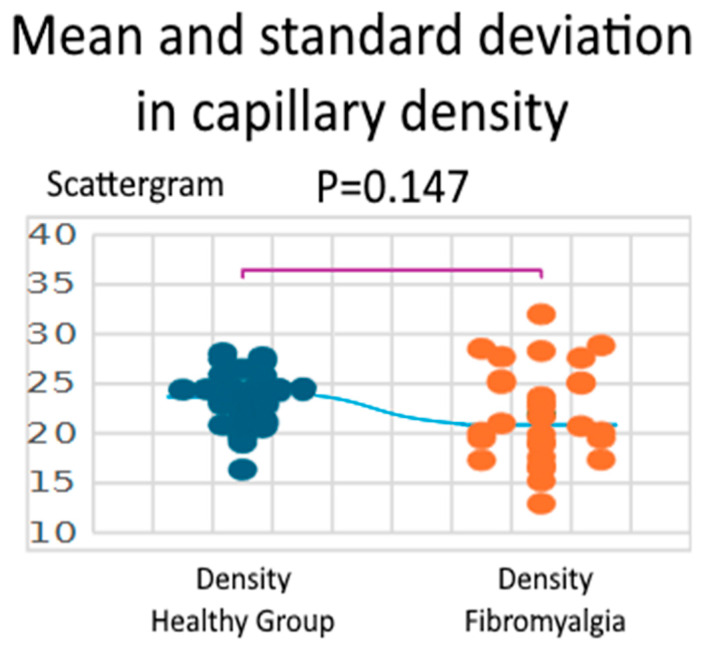
Comparison between healthy group and fibromyalgia Density.

Capillary density did not differ significantly between groups. Healthy controls showed a mean density of 23.37 ± 2.81 loops/mm^2^, while patients with fibromyalgia had a mean of 21.91 ± 4.66 loops/mm^2^. The Shapiro–Wilk test confirmed normal distribution in both groups (W = 0.975, *p* = 0.675 for healthy controls; W = 0.974, *p* = 0.643 for fibromyalgia). The independent two-sample t-test, assuming equal variances, revealed no significant difference between groups (t = 1.469, df = 58, *p* = 0.147; 95% CI: −0.53 to 3.45 loops/mm^2^). While the group difference was not statistically significant, the effect size (Hedges’ g = 0.380) suggests a small-to-medium difference in capillary density (Figure 3).

### 3.3. Length

**Figure 4:**
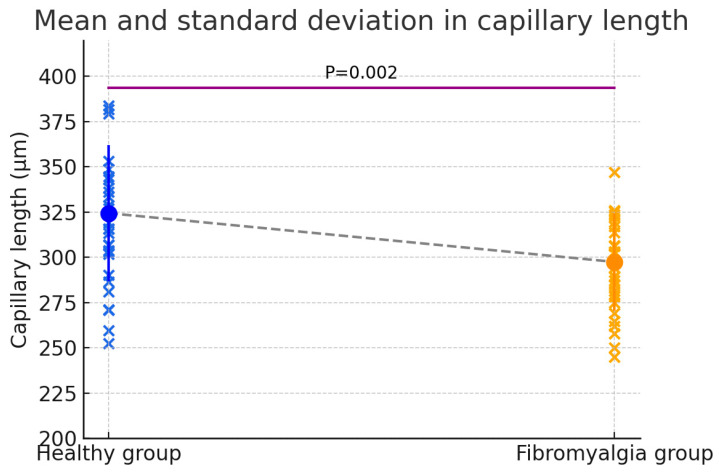
Comparison between healthy group and fibromyalgia Length.

Capillary length was significantly lower in patients with fibromyalgia compared to healthy controls. The mean capillary length was 324.43 ± 37.59 µm in the healthy group and 297.49 ± 26.82 µm in the fibromyalgia group. The Shapiro–Wilk test indicated a non-normal distribution in both groups (W = 0.928, *p* = 0.043 for healthy controls; W = 0.927, *p* = 0.040 for fibromyalgia). The independent two-sample t-test, assuming equal variances, showed a significant difference between groups (t = 3.195, df = 58, *p* = 0.002; 95% CI: 10.06 to 43.81 µm). This significant difference was associated with a large effect size (Hedges’ g = 0.825), indicating a substantial reduction in capillary length in fibromyalgia patients (Figure 4).

### 3.4. Visibility

Fisher’s Exact Test was used to compare the distribution of visibility scores between the two groups. The analysis yielded a *p*-value > 0.05, indicating no statistically significant difference between healthy controls and patients with fibromyalgia (Table 2).

### 3.5. Orientation

The relationship between orientation scores and group status was also assessed using Fisher’s Exact Test. This analysis revealed a statistically significant difference (*p* < 0.05), indicating a meaningful variation in capillary orientation between healthy controls and fibromyalgia patients (Table 3).

### 3.6. Microhemorrhages

No significant difference in the presence of microhemorrhages was observed between healthy subjects and fibromyalgia patients (*p* > 0.05) (Table 4).

In summary, while capillary caliber and density did not differ significantly between groups, capillary length was significantly reduced in fibromyalgia patients. Additionally, qualitative analysis revealed differences in capillary orientation, suggesting detectable microvascular alterations in the oral mucosa associated with fibromyalgia.

## 4. Discussion

In this study, oral mucosal videocapillaroscopy revealed that patients with fibromyalgia exhibited significantly reduced capillary length and altered capillary orientation compared to healthy controls, whereas capillary caliber and density remained unchanged. These findings suggest the presence of subtle microvascular alterations in the oral mucosa of fibromyalgia patients, although their biological and clinical significance remains unclear.

Fibromyalgia is traditionally associated with widespread musculoskeletal pain and fatigue, with the clearest correlations in the oral cavity being related to muscular involvement, such as in cases of temporomandibular disorders [28,29]. However, emerging evidence suggests potential links between fibromyalgia and oral pathologies of inflammatory or autoimmune origin, including Sjögren’s syndrome [30] and oral lichen planus [31], as well as conditions characterized by neural hypersensitivity, such as burning mouth syndrome (BMS) [32]. These conditions are known to present clear microvascular alterations [23,33], raising the question of whether similar, albeit subtler, changes might occur in fibromyalgia.

In our cohort, only capillary length and orientation were significantly affected. Notably, these two parameters are interdependent, as the apparent capillary length can vary depending on its orientation relative to the mucosal surface, with more parallel loops appearing longer and more perpendicular loops appearing shorter. The absence of differences in capillary caliber and density indicates that microvascular alterations in fibromyalgia are likely subtle and may represent early or functional changes rather than overt structural remodeling.

The novelty of this study lies in the application of oral videocapillaroscopy to fibromyalgia, providing preliminary evidence that microvascular features can be detected in this district. Although these changes are not sufficient for diagnostic purposes, they support the hypothesis of systemic microvascular involvement in fibromyalgia. Mechanistically, alterations in endothelial function, low-grade inflammation, and dysregulation of vasoactive mediators such as nitric oxide and endothelin-1 may contribute to these observations.

### Limitations of This Study

The limitations of this Brief Report must be addressed when interpreting the results. These include, first and foremost, the cross-sectional design and the relatively small sample size, which constrain the generalizability of our findings. Second, we acknowledge the lack of strict sex matching between the Fibromyalgia (FM) and Control groups. However, based on the current body of evidence from oral mucosa videocapillaroscopy studies, neither sex nor BMI has ever been reported as a significant discriminant factor for the microvascular parameters analyzed. Notwithstanding this, the lack of strict matching remains a methodological constraint that future, larger studies should address. Third, the presence of uncontrolled confounding variables related to pharmacotherapy (anti-inflammatories, corticosteroids, muscle relaxants) among FM patients is a critical concern. We note that it was neither feasible nor ethical to temporarily cease patient medication for study purposes, as this is a chronic pain condition. Furthermore, due to the highly individualized nature of FM treatment, where no single standardized therapeutic protocol exists and treatment is strongly influenced by the prescribing physician and patient response, it was impossible to create meaningful statistical stratification based on medication usage in this Brief Report. Therefore, the observed differences must be interpreted with caution, recognizing the potential systemic effects of these medications on microcirculation.

## 5. Conclusions

These findings suggest the presence of subtle microvascular changes in the oral mucosa of patients with fibromyalgia. However, given that only two parameters showed statistically significant differences, these alterations are not sufficient for diagnostic purposes or for early detection of the disease. Nevertheless, the results provide preliminary evidence that videocapillaroscopy may capture microvascular features associated with fibromyalgia, supporting the need for further research. Larger studies, ideally with longitudinal follow-up, are warranted to clarify the clinical significance of these observations and to investigate whether such microvascular changes correlate with symptom severity, disease progression, or response to therapy.

## Figures and Tables

**Table 1:** Summary of Parametric Data.

Parameter	Healthy Group	Fibromyalgia	Difference (Fibro—Healthy)	95% CI	t (df = 58)	*p*-Value	Hedges’ g
Caliber (µm)	19.18 ± 0.74 (17.57–20.57)	19.03 ± 0.83 (17.44–20.76)	0.15	−0.26–0.56	0.717	0.476	0.185
Density (n/mm^2^)	23.37 ± 2.81 (16.36–28.09)	21.91 ± 4.66 (12.93–32.00)	1.46	−0.53–3.45	1.469	0.147	0.380
Length (µm)	324.43 ± 37.59 (277.53–417.56)	297.49 ± 26.82 (218.63–374.92)	26.94	10.06–43.81	3.195	0.002 *	0.825

* denotes statistically significant values (p < 0.05).

**Table 2:** Visibility Data.

	Visibility—Healthy	Visibility—Fibromyalgia
1 Clearly	113	110
2 Poorly	5	6
3 Not visible	2	4

**Table 3:** Orientation Data.

	Orientation—Healthy	Orientation—Fibromyalgia
A (Parallel)	113	104
B (Perpendicular)	0	5
AB (Mixed)	5	7

**Table 4:** Microhemorrhages Data.

	Microhemorrhages—Healthy	Microhemorrhages—Fibromyalgia
Yes	1	3
No	117	113

## Data Availability

The raw data supporting the conclusions of this article will be made available by the authors on request.
